# Metabolic Dysregulation in Postmenopause: Implications for Knee Joint Health

**DOI:** 10.3390/jcm14186442

**Published:** 2025-09-12

**Authors:** Ivana Minaković, Jelena Zvekić-Svorcan, Mirjana Smuđa, Bela Kolarš, Darko Mikić, Tanja Janković, Monika Šili, Miljanka Vuksanović, Nevena Đukić, Vesna Mijatović Jovin

**Affiliations:** 1 Faculty of Medicine, University of Novi Sad, 21000 Novi Sad, Serbia; jelena.zvekic-svorcan@mf.uns.ac.rs (J.Z.-S.); mirjana.smudja@uns.ac.rs (M.S.); bela.kolars@mf.uns.ac.rs (B.K.); tanja.jankovic@mf.uns.ac.rs (T.J.); 903012d23@mf.uns.ac.rs (M.Š.); 2 Health Center “Novi Sad”, 21000 Novi Sad, Serbia; 3 Special Hospital for Rheumatic Diseases Novi Sad, 21000 Novi Sad, Serbia; 4 Department of Higher Medical School, The Academy of Applied Studies Belgrade, 11000 Belgrade, Serbia; 5 Medical Faculty of the Military Medical Academy, University of Defence, 11000 Belgrade, Serbia; darenije@gmail.com; 6 Pathology and Forensic Medicine Institute, 11000 Belgrade, Serbia; 7 Faculty of Medicine, University of Belgrade, 11000 Belgrade, Serbia; miljanka.vuksanovic@med.bg.ac.rs; 8 Department of Endocrinology, Clinical Hospital Center Zvezdara, Internal Clinic, 11000 Belgrade, Serbia; 9 Institute for Pulmonary Diseases of Vojvodina, 21204 Sremska Kamenica, Serbia; nevenadjukic.nd@gmail.com; 10 Department of Pharmacology, Toxicology and Clinical Pharmacology, Faculty of Medicine, University of Novi Sad, 21000 Novi Sad, Serbia; vesna.mijatovic@mf.uns.ac.rs

**Keywords:** osteoarthritis, metabolic syndrome, knee joint, radiographic damage, functional impairment, postmenopausal women

## Abstract

**Background/Objectives**: Osteoarthritis is a slowly evolving joint disorder defined by cartilage degradation, synovial inflammation, subchondral bone hardening, and the growth of osteophytes. Increasing evidence highlights the role of metabolic factors in osteoarthritis onset and progression. This study investigated the link between metabolic syndrome and the level of knee impairment in postmenopausal respondents suffering from knee osteoarthritis. **Methods:** A total of 200 participants aged 60–75 years with knee pain were enrolled in this observational cross-sectional study conducted between 2022 and 2023. The case group comprised 120 women with radiographically verified knee osteoarthritis (Kellgren–Lawrence grades II–IV), while 80 age-matched women without radiographic changes served as controls. Clinical and anthropometric measures, metabolic indicators, and radiographic findings were collected. Functional status was assessed using the Lower Extremity Functional Scale and the Lequesne Index. **Results:** The groups differed significantly with respect to the presence of metabolic syndrome, diastolic blood pressure, and fasting glucose level (*p* < 0.05). The metabolic syndrome showed modest but significant associations with radiographic knee damage (effect size 4.7%). After adjusting for smoking status and physical activity level, metabolic syndrome remained significantly associated with radiographic damage (effect sizes: 4.8 and 2.2%, respectively). Participants with osteoarthritis but without metabolic syndrome had better functional knee status compared to those with metabolic syndrome (*p* < 0.05). **Conclusions:** In postmenopausal women, metabolic syndrome is independently associated with radiographic knee damage and contributes to poorer functional outcomes in participants with knee osteoarthritis, underscoring its potential role as a modifiable risk factor.

## 1. Introduction

Osteoarthritis (OA) is a prevalent and progressive musculoskeletal disorder characterized by the gradual degeneration of articular cartilage, inflammation of the synovial membrane, subchondral bone remodeling, and the formation of osteophytes, ultimately resulting in joint dysfunction and structural alterations [1]. The disease arises from a multifactorial interplay involving advancing age, occupational and mechanical loading, previous joint injuries, excess body weight, sex, and hereditary susceptibility [2]. The disease clinically manifests as joint pain, restricted mobility, deformity, and crepitus, which in turn impair daily functioning, diminish quality of life [3,4,5,6] and contribute to a substantial socioeconomic burden [7].

Metabolic syndrome (MetS) is a cluster of interrelated metabolic disturbances, including central obesity, impaired insulin sensitivity, abnormal lipid levels, and elevated blood pressure (BP), which together contribute to systemic low-grade inflammation and adverse cardiovascular and musculoskeletal outcomes [4,8]. Growing evidence suggests that MetS may play a role in the initiation and progression of OA through both metabolic and inflammatory pathways, raising interest in its potential contribution to joint degeneration [9].

Women are disproportionately affected by OA, experiencing not only a higher frequency of the disease but also greater symptom severity and functional impairment, particularly after age 50 [9,10,11]. This increase coincides with the menopausal transition, during which hormonal alterations, declines in bone mineral density and muscle mass, and changes in pain sensitivity may collectively accelerate OA development [12,13]. Estrogens are proposed to exert protective effects on joint tissues by modulating inflammation and stimulating extracellular matrix production in chondrocytes; however, the precise role of estrogen deficiency in postmenopausal OA remains unclear [14,15].

Importantly, menopause is also linked to a substantially higher risk of developing MetS, which may help explain the connection between metabolic dysfunction and the greater prevalence of knee OA in women. Several components of MetS, including visceral adiposity, systemic inflammation, insulin resistance, and adverse lipid profile changes, become more pronounced during the menopausal transition and may further exacerbate structural joint deterioration [4,9].

Despite these observations, the interaction between MetS and structural knee joint changes in postmenopausal women with OA remains insufficiently investigated. Addressing this gap, the present study aimed to examine the association between MetS and the severity of knee joint alterations in older women with knee OA, with the hypothesis that MetS would be positively associated with more advanced structural degeneration.

## 2. Materials and Methods

### 2.1. Subjects

This observational cross-sectional study was conducted at the Special Hospital for Rheumatic Diseases Novi Sad, Serbia, between 2022 and 2023. The study included 200 postmenopausal women presenting with knee pain. Ethical approval was obtained from the hospital’s Ethics Board (approval No. 14/29-3/1-21, issued on 14 September 2021) and from the Clinical Trials Ethics Committee of the Faculty of Medicine, University of Novi Sad (approval No. 01-39/109/1, dated 29 October 2021).

Consecutive female patients who were referred to a physical medicine and rehabilitation specialist with subspecialty training in rheumatology for outpatient evaluation of knee pain were included in the study. The case group was composed of 120 participants with radiographically confirmed OA, classified as Kellgren–Lawrence (KL) grades II through IV. To ensure balanced representation across severity levels, recruitment for the case group was stratified by radiographic grade, with 40 patients enrolled in each of the KL II, KL III, and KL IV subgroups. Recruitment was discontinued once the quota for each subgroup had been reached. The control group was composed of 80 age-matched participants who reported knee pain rated ≥3 on a Numeric Rating Scale and who were subsequently confirmed to have no radiographic evidence of OA (KL grade 0 or I). All assessments for both groups were conducted at the same outpatient facility and under identical evaluation protocols, thereby ensuring that assessment conditions were uniform.

Prior to enrollment, detailed information about the study was provided to all participants, and written informed consent was obtained.

### 2.2. Eligibility Criteria

Eligible participants were women aged 60–75 years with persistent knee pain (≥3 on the Numerical Rating Scale) for at least three months and without a history of estrogen therapy.

The exclusion criteria were as follows: history of knee surgery or trauma, inflammatory rheumatic diseases, or neuromuscular conditions; recent use of corticosteroid therapy—either systemic or intra-articular—within three months prior to enrollment; intra-articular chondroprotective therapy within the last six months; or physical therapy completed less than three months before inclusion. Participants who had experienced changes in the type or dosage of medications for chronic conditions in the previous three months, particularly those affecting carbohydrate, lipid, or cardiovascular metabolism, were also excluded. Malignant diseases constituted an exclusion criterion for both case and control groups. Endocrine disorders were not formally excluded; however, no cases of hyperthyroidism were observed, and a few participants were receiving stable levothyroxine therapy for well-regulated hypothyroidism. Some participants had osteoporosis and were undergoing appropriate treatment.

### 2.3. Subjects’ Evaluation

General health characteristics were documented, including the use of pharmacological treatments for managing hypertension, diabetes, and dyslipidemia. Physical examination included precise measurements of body height, body mass, Body Mass Index (BMI), waist circumference, and BP. Body mass and height were measured using a single standardized device (Birotehna, Smederevo, Serbia) equipped with a digital scale and integrated stadiometer, with an accuracy of ±0.1 kg and ±0.1 cm, respectively. BMI was calculated as the ratio of body mass in kilograms to the square of height in meters (kg/m^2^). Waist circumference was measured with participants in an upright position, during normal breathing, with relaxed abdominal muscles, at the anatomical midpoint between the superior border of the iliac crest and the inferior margin of the rib cage.

Resting BP was measured on both upper limbs using a precisely calibrated and thoroughly verified Riester sphygmomanometer (Jungingen, Germany). Participants were seated upright during the measurements. A second reading was then recorded on the arm that showed the higher BP in the first assessment. The final systolic and diastolic BP values were calculated by averaging the two measurements [16].

Laboratory assessments included triglycerides (mmol/L), fasting glucose (mmol/L), and High-Density Lipoprotein (HDL) cholesterol levels (mmol/L). MetS was diagnosed according to the International Diabetes Federation (IDF) criteria [17]. These criteria require elevated waist circumference as a mandatory component, along with at least two of the following factors: elevated triglycerides ≥ 150 mg/dL (1.7 mmol/L) or specific treatment; reduced HDL cholesterol < 50 mg/dL (1.29 mmol/L) in women or specific treatment; elevated BP—systolic ≥ 130 mmHg or diastolic ≥ 85 mmHg—or previously diagnosed hypertension; and elevated fasting plasma glucose ≥ 100 mg/dL (5.6 mmol/L) or a history of type 2 diabetes mellitus. When BMI exceeds 30 kg/m^2^, central obesity is assumed, and waist circumference measurement is not required. Waist circumference thresholds may vary across ethnic and regional populations, and adjustments may be needed for accurate risk assessment. In this study, a threshold of 80 cm was used for Europid women, in accordance with IDF criteria [17].

Conventional radiographs of both knees were obtained using standard radiographic equipment (DuraDiagnost F30, Philips, Amsterdam, The Netherlands). All images were independently reviewed by the same experienced radiologist, who assessed the extent of knee damage. Radiographic findings were interpreted according to the KL grading system [18] and supplemented by the Altman Atlas for OA evaluation [19]. Definitions of OA grades according to KL criteria are presented in Table 1.

Clinical symptoms and knee function were assessed using the Lower Extremity Functional Scale (LEFS), which has been validated and cross-culturally adapted for the Serbian-speaking population [5]. Functional impairment of the knee was also evaluated using the Lequesne Index [20].

The LEFS consists of 20 questions answered on a 0–4 Likert scale, with higher scores indicating better functional ability. The total score ranges from 0 to 80, providing a quantitative measure of lower extremity function [5,21].

The Lequesne Index includes three subscales. The first subscale (5 items) evaluates pain and discomfort at night, on rising, during prolonged standing, with postural changes, and morning stiffness duration. The second subscale (2 items) assesses maximum walking distance, including pain and the use of assistive devices. The third subscale (4 items) examines daily living activities such as stair climbing, squatting, and walking on uneven ground. Total scores range from 0 (best function) to 24 (worst function) [20].

### 2.4. Statistical Assessment

In accordance with the study’s objectives, the sample size was determined based on a 90% confidence level. This corresponded to a statistical power of 90%, a maximum margin of error of 10%, and an assumed critical incidence rate of 50%. Based on these parameters, a minimum of 80 participants per group was required. Variations among respondents were examined using chi-square tests, with all inferential tests evaluated at a significance threshold of *p* < 0.05. The relationship between MetS and the degree of radiographic knee damage was analyzed using ANCOVA. Differences in knee function status between knee OA patients with and without MetS were tested using an independent *t*-test. All data processing was performed using IBM SPSS Statistics (v25, IBM Corp., Armonk, NY, USA).

## 3. Results

### 3.1. Frequency of MetS and Its Distinct Components

The distribution of MetS and its individual components across the study groups is presented in Table 2. The prevalence of MetS was higher in the case group compared to the control group (81.67% vs. 68.75%). Among individual components, a significantly higher proportion of participants in the case group had diastolic BP ≥ 85 mmHg (30.83% vs. 15.0%, *p* = 0.011) and elevated fasting glucose levels (≥5.6 mmol/L) (74.17% vs. 60.0%, *p* = 0.035). Waist circumference, triglyceride levels, HDL cholesterol, and systolic BP were similar between the two groups.

Differences between the case and control groups were observed in BMI, smoking status, and physical activity level. The case group had a higher mean BMI 31.34 (SD = 5.20) than the control group 28.97 (SD = 4.89), and this difference was statistically significant (*p* = 0.001). No significant differences were found between the groups regarding smoking status (current, former, or non-smokers) (*p* = 0.380). In contrast, a significant difference was observed in physical activity level, with a higher proportion of participants in the case group reporting low activity compared to moderate activity (*p* < 0.001). None of the participants reported engaging in high-intensity physical activity (Table 3).

### 3.2. The Relationship Between Mets and Knee Joint Impairment

The impact of MetS, defined according to the IDF criteria, on the degree of radiological knee damage was assessed using ANCOVA (Analysis of Covariance). MetS significantly influenced the extent of radiological damage (*p* = 0.002), accounting for 4.7% of the variance as indicated by eta squared. This association remained statistically significant after adjusting for smoking status and physical activity. Adjustment for BMI was not performed because BMI and waist circumference were found to be highly correlated, as indicated by VIF and tolerance values, and including both variables could introduce multicollinearity (Tolerance = 0.18) (Table 4).

**Figure 1:**
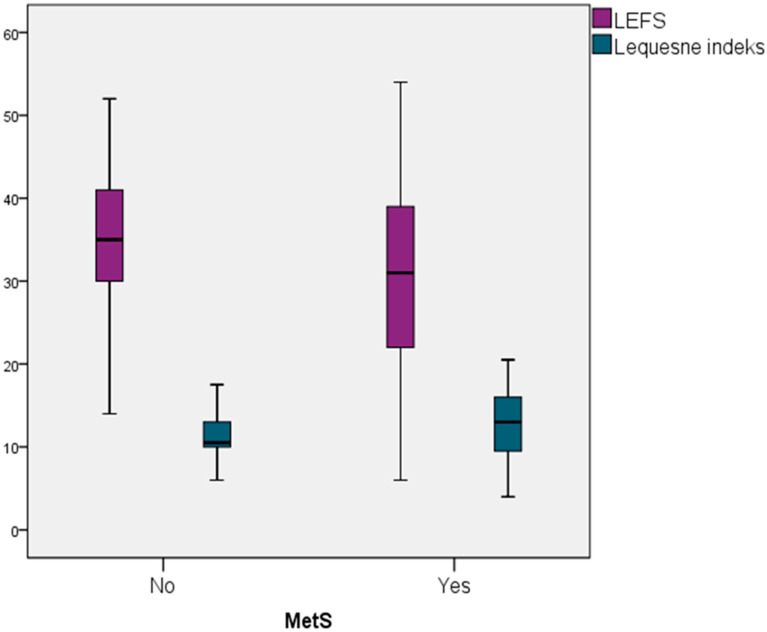
Difference in Knee Function Status Between Knee OA Patients With/Without MetS.

In participants with knee OA (case group), those without MetS had a higher mean LEFS score compared to those with MetS 34.77 (SD = 8.60) vs. 29.74 (SD = 10.78), and this difference was statistically significant (*p* = 0.032). Similarly, participants with MetS exhibited higher mean Lequesne index scores than those without MetS (12.82 (SD = 3.84) vs. 11.23 (SD = 3.22)) and this difference was also statistically significant (*p* = 0.036). These results are illustrated in a box plot 1 (Figure 1).

## 4. Discussion

The initial hypothesis of this study was that MetS would be positively associated with the severity of structural knee changes in postmenopausal women, suggesting a possible metabolic contribution to joint degeneration. OA is a chronic degenerative condition more prevalent among older adults, individuals with obesity, and females, commonly presenting with joint pain, stiffness, and reduced function. While current treatments primarily alleviate symptoms, they have limited impact on joint pathology, often making arthroplasty the final option in advanced cases, highlighting the importance of understanding modifiable risk factors for prevention [22]. The underlying causes of OA remain incompletely clarified, with emerging research indicating that it is a multifactorial condition driven by diverse pathological mechanisms [23,24]. Although aging and obesity are recognized as significant determinants for OA onset, disruptions in metabolic homeostasis are also thought to contribute substantially to its development [23,25,26]. In individuals with obesity, excessive loading of the lower limbs may result from a combination of factors, including increased weight-bearing during walking, altered gait patterns due to irregular body mass distribution, and accompanying systemic inflammation [27].

The findings of our study indicate that the presence of MetS according to IDF criteria [17] exhibit a modest but statistically significant association with the degree of radiographic knee damage, contributing 4.7% to the explained variance. The association between MetS and radiographic knee damage remained statistically significant even after adjusting for confounding factors such as smoking status and physical activity level. Additionally, among participants with radiographically confirmed OA, those diagnosed with MetS exhibited significantly lower LEFS scores and higher Lequesne Index values, indicating that poorer knee function is associated with the presence of MetS.

Evidence from large-scale studies indicates that individuals with chronic obesity are particularly susceptible to medial knee cartilage damage, which contributes to OA progression [28]. In a cohort of over 1.7 million participants, the incidence of knee OA was found to be more than three times higher in obese individuals compared to those with normal body mass [29]. Furthermore, each 5 kg/m^2^ increase in BMI has been associated with a 35% higher risk of developing knee OA [30].

When examining the relationship between MetS and OA, a critical question arises: do metabolic disturbances associated with obesity represent independent risk factors for OA [31]? Although several studies have reported significant associations between MetS and OA, the findings remain inconsistent [32,33,34]. A systematic review which included 40 studies indicated a positive association between radiographically confirmed OA and MetS, hypertension, and hyperglycemia [22], whereas findings from a large longitudinal cohort of over half a million subjects showed that MetS was linked to a 15% higher likelihood of developing OA [35]. Furthermore, pooled data from eight epidemiological studies revealed that individuals with MetS face a greater risk of developing both symptomatic and radiographic knee OA [32]. In contrast, other systematic reviews have reported no clear association between MetS and OA, nor evidence that MetS contributes to an elevated risk of OA development [33,34]. Notably, although adjusted analyses from a meta-analysis of five prospective studies found no significant association between MetS and the overall risk of knee OA, further subgroup evaluation revealed that MetS independently contributes to an elevated risk of developing more advanced stages of knee OA, while showing no clear link to symptomatic cases in general [33]. Supporting this observation, findings from a recent five-year Dutch follow-up study suggest that women exhibiting more pronounced MetS, as reflected by elevated z-scores, may face a greater risk of structural progression in knee OA compared to those with less severe metabolic profiles [36].

Longitudinal studies examining hip or knee arthroplasty as the primary outcome have consistently reported no significant association between MetS and the risk of hip arthroplasty [37,38]. In contrast, findings from a large Australian cohort study of 20,430 individuals showed that MetS was linked to over a threefold higher likelihood of undergoing knee replacement surgery, independent of BMI [11].

Regarding the relationship between MetS and knee function, consistent with our findings Samaan at al [39]. reported that the coexistence of knee OA and MetS is linked to greater functional impairment and reduced quality of life. Additionally, a recent study involving over 6000 participants found that participants with MetS experienced greater pain, reduced quality of life, and slower walking speed compared to participants without metabolic risk factors, suggesting that MetS may hinder rehabilitation in OA [40]. However, a population-based analysis indicates that this association may be largely mediated by BMI [41].

To isolate the metabolic effects from the biomechanical impact of obesity, statistical adjustments excluding the influence of BMI are commonly employed [31,33,42]. However, the strong correlation between central obesity (a predominant component of MetS) and BMI, along with the limitations of BMI as a proxy for joint load, raises methodological concerns that remain unresolved [43]. The imprecise use of BMI in this context stems from its inability to differentiate between fat and lean mass, as it simply reflects body mass relative to height (kg/m^2^) [36,43]. BMI does not account for body fat distribution or distinguish adipose tissue from muscle mass. While increased muscle mass is considered protective against knee OA, higher fat mass is a recognized risk factor. Given that both BMI and body fat percentage are causally linked to the development and progression of knee OA, body fat percentage may serve as a more accurate indicator than BMI [3].

In addition to the above, the inconsistent findings concerning the impact of MetS on the development and advancement of OA can be attributed to considerable differences in research framework, criteria for disease identification, data analysis methods, and the demographic features of the populations examined [31,34]. Importantly, definitions of OA have varied across studies—from self-reported and clinically diagnosed cases to severe symptomatic OA requiring knee replacement as the final outcome. Diagnostic inconsistency in defining MetS leads to substantial variation in its reported prevalence and that of its components. This discrepancy is largely attributable to differing threshold values—most notably for waist circumference and fasting glucose, with cut-offs of 5.6 mmol/L in the IDF criteria and 6.1 mmol/L in the NCEP definition [17,44].

This study has several strengths, including a well-defined and homogeneous sample of postmenopausal women, consistent radiographic evaluation by a single experienced radiologist, and the use of both structural (KL grading) and functional (LEFS, Lequesne index) outcome measures. Importantly, our analyses were adjusted for relevant confounding variables such as smoking status and physical activity, which strengthens the reliability of the observed associations.

However, certain limitations should be acknowledged. The cross-sectional design does not allow for conclusions regarding causality. Some data, including lifestyle factors, were self-reported and thus subject to recall bias. The study was conducted at a single specialized center, which may limit generalizability. Additionally, structural assessment was based solely on radiographic findings, which may not capture early joint changes detectable by more advanced imaging techniques.

## 5. Conclusions

Our findings indicate that MetS contributes both to structural joint damage and to diminished knee function among postmenopausal women with OA. Recognizing the metabolic dimension of OA may open opportunities for more comprehensive prevention and treatment approaches, which should be further explored in future prospective studies.

## 6. Practical Implications

The observed associations between MetS and both structural joint changes and poorer functional outcomes in postmenopausal women with knee OA highlight the need for a broader, integrated approach to disease management. Given its impact on both structural joint changes and functional status, metabolic health represents a key target for interventions. Strategies addressing obesity, hypertension, dyslipidemia, diabetes, and lifestyle factors such as diet and physical activity could meaningfully improve symptom burden and overall quality of life. Incorporating routine screening and management of metabolic risk factors alongside conventional OA therapies may help slow progression and enhance patient outcomes. Future research should examine whether integrated metabolic interventions can attenuate structural and functional decline and potentially delay disability in this vulnerable population.

## Figures and Tables

**Table 1:** KL Classification of Osteoarthritis.

Grade	Description
0	No radiographic features of osteoarthritis are present.
1	Doubtful joint space narrowing and possible osteophytic lipping (tiny bone growths).
2	Definite osteophytes and possible joint space narrowing on anteroposterior weight-bearing radiograph.
3	Multiple osteophytes, definite joint space narrowing, sclerosis, and possible deformity of bone contour.
4	Large osteophytes, marked joint space narrowing, severe sclerosis, and definite deformity of bone contour.

KL = Kellgren–Lawrence grading system [18].

**Table 2:** MetS and Its Individual Components, Differences Between Groups.

		All (*n* = 200)	Case Group (*n* = 120)	Control Group (*n* = 80)	*p*
WC, *n* (%)	<80 cm	23 (11.5%)	12 (10.0%)	11 (13.75%)	0.415
	≥80 cm	177 (88.5%)	108 (90.0%)	69 (86.25%)	
Triglycerides, *n* (%)	<1.7 mmol/L	110 (55.0%)	68 (56.67%)	42 (52.5%)	0.562
	≥1.7 mmol/L	90 (45.0%)	52 (43.33%)	38 (47.5%)	
HDL cholesterol, *n* (%)	<1.29 mmol/L	42 (21.0%)	26 (21.67%)	16 (20.0%)	0.777
	≥1.29 mmol/L	158 (79.0%)	94 (78.33%)	64 (80.0%)	
Systolic BP, *n* (%)	<130 mmHg	96 (48.0%)	51 (42.5%)	45 (56.25%)	0.057
	≥130 mmHg	104 (52.0%)	69 (57.5%)	35 (43.75%)	
Diastolic BP, *n* (%)	<85 mmHg	151 (75.5%)	83 (69.17%)	68 (85.0%)	0.011
	≥85 mmHg	49 (24.5%)	37 (30.83%)	12 (15.0%)	
Fasting glucose, *n* (%)	<5.6 mmol/L	63 (31.5%)	31 (25.83%)	32 (40.0%)	0.035
	≥5.6 mmol/L	137 (68.5%)	89 (74.17%)	48 (60.0%)	
MetS, *n* (%)	Yes	153 (76.5%)	98 (81.67%)	55 (68.75%)	0.035

WC = waist circumference, HDL = High-Density Lipoprotein, BP = Blood Pressure, MetS = Metabolic Syndrome, *n* = number of participants, % = percentage, *p* = statistical significance. Chi-square tests were performed.

**Table 3:** BMI, Smoking status, Physical activity, and Differences Between Groups.

		Case Group (*n* = 120)	Control Group (*n* = 80)	*p*
BMI, M (SD)		31.34 (5.20)	28.97 (4.89)	0.001 ^a^
Smoking status, *n* (%)	Current smoker	33 (27.5%)	20 (25.3%)	0.380 ^b^
	Former smoker	23 (19.2%)	10 (12.7%)	
	Non-smoker	64 (53.3%)	49 (62.0%)	
Physical activity, *n* (%)	Low	83 (69.7%)	23 (29.1%)	˂0.001 ^b^
	Moderate	36 (30.3%)	56 (70.9%)	

BMI = Body Mass Index, M (SD) = Mean (Standard Deviation), *n* = number of participants, % = percentage, *p* = statistical significance. ^a^ Independent *t*-test, ^b^ Chi-square tests.

**Table 4:** The relationship between MetS with the degree of radiographic knee damage.

	df	Mean Square	F	*p*	Partial Eta^2^
MetS	1	12.737	9.727	0.002	0.047
MetS (adjusted for Smoking status)	1	13.046	9.938	0.002	0.048
MetS (adjusted for Physical activity)	1	4.732	4.469	0.036	0.022

MetS = Metabolic Syndrome, F = ANCOVA test, df = degrees of freedom, *p* = statistical significance.

## Data Availability

The original contributions of this research are fully presented within this article. For any further information or clarification, interested parties are encouraged to contact the corresponding author. The data underlying the findings of this study can be accessed by contacting the corresponding author, provided that the request is justified; however, public sharing is restricted to protect ethical standards and maintain participant confidentiality.

## Abbreviations

The following abbreviations are used in this manuscript:

OA	Osteoarthritis
MetS	Metabolic Syndrome
BP	Blood Pressure
KL	Kellgren–Lawrence
BMI	Body Mass Index
HDL	High-Density Lipoprotein
IDF	International Diabetes Federation
LEFS	Lower Extremity Functional Scale
